# Antioxidants and Prooxidants: Effects on Health and Aging 2018

**DOI:** 10.1155/2019/7971613

**Published:** 2019-02-28

**Authors:** Márcio Carocho, Isabel C. F. R. Ferreira, Patricia Morales, Marina Soković

**Affiliations:** ^1^Centro de Investigação de Montanha (CIMO), Instituto Politécnico de Bragança, Portugal; ^2^Faculty of Pharmacy, Complutense University of Madrid, Spain; ^3^Institute of Biological Research, University of Belgrade, Serbia

In the mid 50's, a ground-breaking theory was proposed by Denham Harman, which revolutionized the understanding of biology and aging and to an extent helped shape the research that was put forward to understanding the mechanisms of growing old and the underlying workings at a cellular level [1]. This theory, known as the “Free Radical Theory of Aging,” is the cornerstone of this special issue; health is the most important feature that a human being wishes for, although conscient of the demise of the body, conscient of growing old. Over the years, the theory has seen some tough arguments against it and others that back its overall postulations.

By 1992, after nearly 40 years of scientific advancements, Denham Harman wrote that “the data supporting this theory indicates that average life expectancy at birth may be increased by 5 or more years, by nutritious low-calorie diets supplemented with one or more free radical reaction inhibitors,” paving the way for the increased incidence of research on plants, fruits, and vegetables, as possible “radical reaction inhibitors” when consumed regularly in the diet could help increase the 5 years of life expectancy. Still, D. Harman at the time understood that aging could be explained by many theories, namely, molecular cross linking, changes in immunological function, and the senescence genes in the DNA [2]. 40 years after its appearance, Beckman and Ames [3] wrote that the theory had matured and systematically revised the findings that had been brought forward in terms of the implications of free radicals, prooxidants, and aging. During the early 2000's, the theory was supported by many evidences that free radicals could explain recently discovered phenomena, although definitive proof that oxidised molecules were the primary cause of aging was lacking confirmation [4]. These revelations reduced the momentum the theory had been gaining in the previous decades. By 2014, Vadim Gladyshev had “killed” the theory, arguing that although Reactive Oxygen Species (ROS) were by-products responsible for cellular damage, their contribution to aging was governed by cellular metabolic organization, protective systems, and the individual's genotype. Furthermore, oxidative damage, individually or in combination, could not represent the cause of aging [5].

Even though its death had been predicted in 2014, the truth is that even though its name has been changed and its basic premises altered, the theory continues to inspire many researchers across the globe to find the mechanisms of health and aging. By 2018, “A Free Radical Theory of Frailty” was published, stating that oxidative damage correlates not with chronological age but with the human frailty state [6]. Still, even if researchers digress from the original theory and improve its reasonings, free radicals, oxidative stress, anti- and prooxidants, aging, and health are linked and are still as important to research today as they were 60 years ago.

Roughly a year ago, in our first editorial (2017) of the special issue “Antioxidants and Prooxidants: Effects on Health and Aging” [7], we wrote that the issue intended to shed some light on the dichotomy of antioxidants and prooxidants, given the important role that either have on the maintenance of health and their contribution to how aging unfolds. One year after, this 2018 edition aims at exactly the same outcome, to dwell deeper in the understanding of this inevitable happening that is called aging. Today, even though mankind is closer, it still is not clear why we age, albeit the theories that have been postulated though the years. So, this quest for the answers should be the driving force to increase research on human health and aging, to reach the ultimate goal of knowing why we gradually lose our health and ultimately meet our demise.

This year's edition, comprised of 15 articles, focuses on many aspects of antioxidant and prooxidant activities, with authors from 9 distinct countries, namely, Russia, China, Czech Republic, Austria, Pakistan, Mexico, Greece, Italy, and Poland. The study types are quite diverse, with focus on *in vitro cell* lines both from human tumours and various mice cell lines, studies on rabbits, mice, rats, and two with humans, namely, athletes and professional football players. In this edition, there are some interesting articles; two of them report the antioxidant activity of natural compounds, namely, curcumin and ellagic acid. Fullerene derivatives are studied for their effectiveness against human lung fibroblasts while pyrrolidine dithiocarbamate is used to inhibit deoxynivalenol damage to the mitochondria. This same organelle is also targeted by isoliquiritigenin in another study. Antioxidant enzymes are also an important part of the special edition, with two articles devoted to their study. One focuses on the alleviation of intervertebral disc degeneration by modulating antioxidant enzymes with estradiol, while the other focuses on the consequences of impairment of aldehyde dehydrogenase. The effect of food in the redox status of elite football players is also researched, in this case, through the consumption of dark chocolate. Another study involving human studies, namely, athletes, in which the effect of exercise induced reductive stress is assessed as a protective mechanism against oxidative stress in blood mononuclear cells. Finally, the supply of zinc in a chronic cadmium environment is viewed in the sublingual gland structure. We hope that these articles are of interest to the readers of this special edition and may help the progress of science.
